# Inhibition of Autophagy in Microglia Alters Depressive-Like Behavior via BDNF Pathway in Postpartum Depression

**DOI:** 10.3389/fpsyt.2018.00434

**Published:** 2018-10-08

**Authors:** Xiaoning Tan, Xiaoxue Du, Yuting Jiang, Benson O. A. Botchway, Zhiying Hu, Marong Fang

**Affiliations:** ^1^Institute of Neuroscience, Zhejiang University School of Medicine, Hangzhou, China; ^2^Department of Obstetrics and Gynecology, Hangzhou Red Cross Hospital, Hangzhou, China

**Keywords:** microglia, fluoxetine, inflammation, chronic unpredicted mild stress, brain-derived neurotrophic factor (BDNF)

## Abstract

Postpartum depression (PPD) is associated with mood disorders and elevated inflammation. Studies have evidenced the activation/inhibition of autophagy and excessive activation of microglia to have a close relationship with depression. C57 and microglia-specific autophagy-deficient mice (Cx3Cr1^Cre/+^ATG5^loxp/loxp^) were employed to establish the chronic unpredicted mild stress depression mice model from embryonic day 7 (E7) to embryonic day 16 (E16). Fluoxetine was administered for 3 weeks (commencing from 1 week after birth). Behavioral tests (open field, forced swimming, and sucrose preference tests) were implemented. Western blot and immunofluorescence staining were employed to assess the brain-derived neurotrophic factor (BDNF) expression level, autophagy-associated proteins, and inflammatory factors. Depressive behavior was reversed following fluoxetine treatment; this was evidenced via open field, sucrose preference, and forced swimming tests. Both BDNF and autophagy-associated proteins (ATG5, Beclin-1, and LC3II) were upregulated following fluoxetine treatment. Inflammatory factors including nuclear factor kappa B and inducible nitric oxide synthase were reduced while anti-inflammatory factor interleukin-10 (IL-10) was increased after fluoxetine treatment. Microglia-specific autophagy-deficient mice (Cx3Cr1^Cre/+^ATG5^loxp/loxp^) showed a curtailed autophagy level, higher inflammatory level, and reduced BDNF expression when compared with C57 mice. Autophagy inhibition in microglia contributes to inflammation, which further instigates PPD. Fluoxetine might mediate its antidepressant effect in PPD through the autophagic pathway while upregulating BDNF expression. In view of this, regulating BDNF in microglia is a potential novel therapy target for PPD.

## Introduction

Postpartum depression (PPD) is often accompanied by extreme sadness and hopelessness, low energy, anxiety, crying episodes, irritability, and infanticide (1, 2). This mental disorder is common, severe, and experienced by 13–19% of new mothers (3, 4). Occurrence of depressive-like behavior following childbirth can be pernicious to mothers, fathers, and possibly children if untreated (5), subsequently leading to devastating outcomes to family and society. Numerous factors such as the interaction effect of genetic and epigenetic susceptibilities combined with environmental risk factors, such as stress, hormonal level change, and emotional trauma may account for the emergence of PPD. Chronic stress during pregnancy is one of the most important causes of PPD. Maternal stress exposure and fluoxetine treatment have detrimental effects on offspring (6). Although neurogenesis stimulation in the hippocampus of depressed mothers following chronic fluoxetine administration in the postpartum period has been demonstrated, the effects of selective serotonin reuptake inhibitors (SSRI) pertinent to reversing stress-induced behavioral, structural, and pathological changes in postpartum females have not been evaluated (7). Presently, there is not a widely convincing mechanistic hypothesis of PPD; nonetheless, the serotonin and neurotrophin hypothesis of depression could partly account for PPD pathology. Brain-derived neurotrophic factor (BDNF) is a well-known neurotrophic factor whose deregulation is closely linked to affective disorders (8, 9). Increased levels of monoamines in relieving depressive symptoms have been evinced; however, there are no convincing theories linking serotonin elevation to increased BDNF transcription and, thus, no elucidation as to how antidepressants such as SSRIs activate the BDNF pathway (10). A large number of antidepressant drugs are already available for PPD treatment, with fluoxetine being one of the most commonly used drugs. Therefore, investigating its effects on PPD pathogenesis is paramount and warranted.

Microglia is a group of neuroglia located in the brain and the spinal cord. As the resident macrophage population of the central nervous system (CNS), microglia act as a first and main guard of active immune defense, closely related to inflammation (11). There are ample evidences indicating the neurotoxicity of overreactive microglia (12, 13). Microglial activation has two main phenotypes: M1 and M2. The M1 phenotype is associated with increments in interleukin-1β (IL-1β) and tumor necrosis factor-α (TNF-α), while the M2 phenotype is associated with the release of anti-inflammatory cytokines such as IL-10 (13). Though studies have shown microglial activation to be evident in psychiatric conditions, authoritative research linking microglial activation to the occurrence of mood disorders is lacking (14). Nonetheless, microglia might be a good target in the development of novel antidepressant drugs for PPD.

Autophagy is an intracellular bulk degradation process responsible for the clearance of damaged proteins and organelles and hence an important regulator of homeostasis and functions in the CNS. An increasing number of studies indicate the relationship between impaired autophagy and affective disorders (15). Autophagy is considered to have evolved as a stress response, interfacing with most cellular stress-response pathways such as immune response and inflammation (16). Autophagy and autophagy-related proteins are essential components that modulate the inflammatory response either directly by acting on the stability or secretion of inflammatory mediators or indirectly by suppressing intracellular stressors (17).

Since the role of autophagy in PPD pathogenesis is ambiguous, this study investigates whether the inhibition of autophagy in microglia arouses inflammation, further influencing the usage of antidepressants in PPD treatment. Considering that the neuroprotective role of the BDNF pathway has been reported in previous studies and combining evidence from other depression studies, we hypothesized that the inhibition of autophagy in microglia may have a detrimental effect on fluoxetine treatment for PPD through upregulation of inflammation and reduced BDNF expression.

## Materials and method

### Animals

Adult female C57 mice and microglia-specific autophagy-deficient mice (Cx3Cr1^Cre/+^ATG5^loxp/loxp^) descripted as ATG5 KO in the following test were used in this experiment. A total of 30 female mice were randomized into six groups. They weighed 20 g ± 2 g and were obtained from Zhejiang Provincial Academy of Medical Sciences. Preceding the experiment, the mice were reared in the experimental animal facility for a week so as to acclimatize them to the new environment. Temperature was set between 24 ± 1°C with humidity at 55% coupled with a 12 h light–dark cycle. Standard food pellets and tap water were made available at all times during the experiments. All experiments were conducted following the National Institutes of Health Guide for the Care and Use of Laboratory Animals. The Ethics Committee for Animal Research at Zhejiang University approved the experimental procedures.

### Drug administration

Fluoxetine (Patheon, France) was dissolved in 0.9% normal saline. Fluoxetine and normal saline were administered from postnatal day 7 (P7) to postnatal day 28 (P28) at a dose of 18 mg/kg/d (18).

### Chronic unpredictable mild stress (CUMS) procedure

CUMS modus operandi was modified from that used by Kiryanova et al. (6). From embryonic day 7 (E7) of pregnancy, dams were subjected to the regimen of CUMS. Embryonic day 16 (E16) was the last day the stressors were administrated (Table 1). Stressors included restraint stress (mice were subjected to chronic-restraint stress by placement in 50 ml conical tubes with holes for air flow for 2 h), restricted access to food (food was removed from animal's house for 6 h), forced swimming (5 min per mouse), continuous lighting overnight, foreign object in cage (a novel plastic object), cage tilting (home cage tilted 30°), white noise (played at 80 dB), and soiled cage (100 ml of clean water spilled on bedding).

**Table 1:** Chronic unpredictable mild stress schedule used on pregnant mouse dams from E7–E16.

	**Day1**	**Day2**	**Day3**	**Day4**	**Day5**
9 a.m.		Restricted access to food			
10 a.m.	Restraint Stress	9 a.m.−3 p.m.		Cage Tilt(30°)	Restricted access to food
11 a.m.	10 a.m.−12 p.m.		Restraint Stress	10 a.m.-5 p.m.	10 a.m.−5 p.m.
12 p.m.		Force Swim	11 a.m.−1 p.m.		
1 p.m.					White noise
2 p.m.					12 p.m.−3 p.m.
3 p.m.					
4 p.m.					
5 p.m.	Paired housing	Continuous lighting	Foreign object in cage	Paired housing	Soiled cage
	5 p.m.−9 p.m.	overnight	5 p.m.−10 a.m.	5 p.m.−10 p.m.	5 p.m.−10 p.m.
	**Day6**	**Day7**	**Day8**	**Day9**	**Day10**
9 a.m.				Restricted access to food	
10 a.m.			Restraint Stress	9 a.m.−3 p.m.	
11 a.m.			10 a.m.−12 p.m.		Restraint Stress
12 p.m.				Force Swim	11 a.m.−1 p.m.
1 p.m.					
2 p.m.					
3 p.m.					
4 p.m.					
5 p.m.	Continuous lighting	Cage Tilt(30°)	Paired housing	Continuous lighting	Foreign object in cage
	Overnight	5 p.m.−10 a.m.	5 p.m.−9 p.m.	overnight	5 p.m.−10 a.m.

### Experiment design

A total of 30 pregnant female mice were randomly assigned to six groups: (1) C57(Normal)+CUMS+ Pregnancy, Fluoxetine (N+C+P-FLX) (*n* = 5); (2) C57+CUMS+ Pregnancy, saline (N+C+P-con) (*n* = 5); (3) C57+ Pregnancy (N+P) (*n* = 5); (4) ATG5KO+CUMS+ Pregnancy, Fluoxetine (A+C+P-FLX) (*n* = 5); (5) ATG5KO+CUMS+ Pregnancy, saline (A+C+P-con) (*n* = 5); (6) ATG5KO+ Pregnancy (A+P) (*n* = 5).

**Figure 1:**
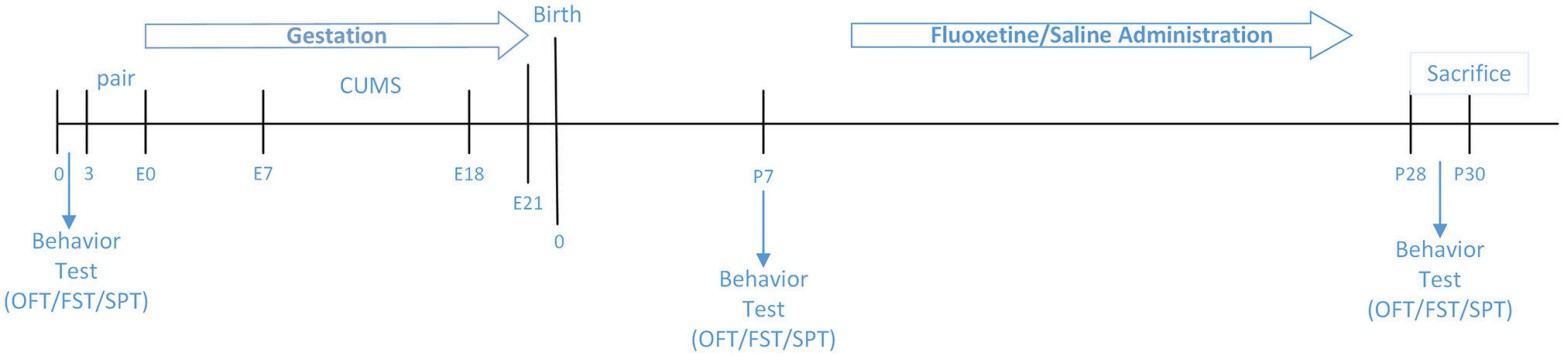
Couse of the study.

Figure 1 illustrates experimental design.

### Behavioral test

#### Forced swimming test (FST)

Mice individually placed in a cylinder of water (24 ± 1°C) swam for 6 min under normal light. Water depth was set to prevent the animals from touching the bottom with their tails or hind limbs. Animal behaviors were videotaped from the side. Immobile time during the last 5 min of the test was evaluated by two observers blinded to animal treatment. Immobile time was considered as the time when the mice remained floating or motionless with only small movements necessary to keep balance in water.

#### Open field test (OFT)

A novel open field environment (comprising 45 cm × 45 cm × 45 cm white Plexiglas arena) was employed to test the exploratory locomotion of mice, with each test lasting 5 min. Each mouse was placed in the center of arena at the beginning and allowed to freely move and its movement was recorded by an overhead camera. The behavior of the mouse was analyzed with an automatic behavior-tracking system (Video Track, Viewpoint Inc., France). Total distance (horizontal activity) and small distance movements as well as the number of rearing (vertical activity) and time spent in the central zone (25 × 25 cm) were recorded (19). The chamber was cleaned with 75% ethanol after each mouse was tested. The ratio of small distance to whole distance was analyzed, depicting exploratory ability.

#### Sucrose preference test (SPT)

Mice were single housed and habituated with two bottles of water for a day, followed by two bottles of 2% sucrose for a day. Following that, mice were water deprived for 23 h and then exposed to one bottle of 2% sucrose and one bottle of water for 2 h in the dark phase (bottle positions switched after 1 h). Total consumption of both water and sucrose were measured, and sucrose preference was recorded as the average sucrose consumption ratio during the first and second hours. The sucrose consumption ratio was calculated by dividing the total consumption of sucrose by the total consumption of both water and sucrose (20).

### Western blot

Total proteins of the hippocampal brain tissue were extracted from each group using ice-cold RIPA buffer, with added protease and phosphatase inhibitors. After grinding with liquid nitrogen and centrifuging at 12,000 rpm for 30 min at 4°C, supernatant proteins were collected and preserved at −80°C in the fridge. The concentration of samples was determined with BCA kits (KeyGEN) and unified to 2μg/μL. SDS-PAGE loading buffer (protein sample:loading buffer = 4:1) was added and the protein sample boiled in 100°C water. A total of 20 μg protein of each sample was subjected to electrophoresis on 15% SDS-PAGE gel using a constant voltage (200 V). Afterwards, the separated proteins were transferred into a polyvinylidene difluoride (PVDF) membrane using Bio-Rad Transblot apparatus. This process was performed under a constant voltage of 100 V for 70 min. PVDF membranes were blocked with 5% skim milk diluted by TBST for 3 h at room temperature. The membranes were then incubated with different antibodies overnight at 4°C. Primary antibodies used were: rabbit polyclonal antibody against GAPDH (Cell Signal Technology, 1:1,000), ATG5/ATG12 (Abcam, 1:1,000), LC3II (NOVUS, 1:1,000), BDNF (Abcam, 1:1000), Beclin-1 (Cell Signal Technology, 1:1,000), inducible nitric oxide synthase (iNOS) (BOSTER, 1:1,00), IL-10 (BOSTER, 1:100), and nuclear factor kappa B (NF-κB) (Cell Signal Technology, 1:1000). After the incubation of primary antibodies, the membranes were washed with TBST thrice, each wash lasting 5 min. Membranes were then incubated with goat antirabbit IgG antibody (BOSTER, 1:5,000) at room temperature for 2.5 h. Washing was done again with TSBT thrice, with each wash lasting 5 min. The membranes were readied for exposure using the ChemiDoc Touch Imaging System after incubating with enhanced chemiluminescence. The grayscale value of each band was analyzed by using the Image Lab program. Each experiment was performed three times.

### Immunofluorescence staining

Mice were anesthetized with 10% chloral hydrate, cardiac perfused with 50 mL (±) 0.9% normal saline to flush their vascular blood, and then perfused with 4% paraformaldehyde in 0.01 M phosphate-buffered saline (PBS, pH 7.4). After perfusion, brain tissue was obtained and conserved in 4% paraformaldehyde for at least 1 day and the fixation fluid replaced with 30% sucrose solution. Embedding and frozen tissue section were performed using a freezing microtome (Leica, Wetzlar, Germany). Frozen sections of 18 μm thick tissue were dried at 37°C for 1 h and then blocked with 5% normal goat serum at room temperature for 1 h. Primary antibodies were applied overnight at 4°C: Iba1 (Abcam, ab178847, 1:100), BDNF (Abcam, 1:1,000), and NeuN (Abcam, 1:1,000). Sections were rinsed and incubated with secondary antibodies, antirabbit or antimouse Alexa Fluor594 (1:500, EARTHOX, USA) in 1% BSA and 0.3% Triton X-100 in PBS for 3 h at room temperature. Sections were then washed again three times with 0.01 M PBS, and a mounting medium containing DAPI (VECTASHIELD, USA) was added to the slides and then covered with coverslips for observation. Slides were observed under a fluorescence microscope (Olympus BX51, NIKON, Japan) at excitation/emission wavelengths of 547/570 nm (Cy3, red), 494/520 nm (FITC, Green), and 360/460 nm (DAPI, blue). Images were taken at 200 × magnification.

### Statistical analysis

Data were analyzed with one-way ANOVA using SPSS 20.0 and histograms were generated in GraphPad Prism 5. Data for behavioral test, western blotting, and immunofluorescence were expressed as mean ± SEM. Gray values of western blot results were calculated by using Image Lab software. Immunofluorescence results of Iba1 were analyzed by using Pro Image Plus. All results were considered statistically significant at ^*^P < 0.05, ^#^*P* < 0.05, ^∧^*P* < 0.05, ^**^P < 0.01, ^##^*P* < 0.01, ^∧∧^*P* < 0.01, ^&&^*P* < 0.01, ^***^P < 0.001, and ^∧∧∧^*P* < 0.001.

## Results

### CUMS induces depressive-like behaviors

**Figure 2:**
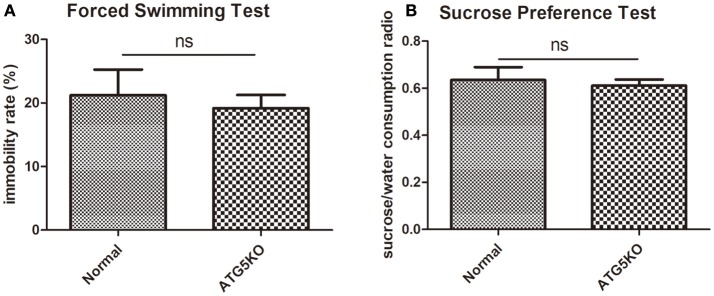
Baseline behaviors of normal and ATG5KO mice before chronic unpredictable mild stress (CUMS) exposure. **(A)** Immobility rate of normal and ATG5 KO groups in forced swimming test (FST). **(B)** Sucrose/water consumption ratio of normal and ATG5 KO groups in sucrose preference test (SPT). Values are expressed as mean ± SEM (*n* = 5). Normal vs. ATG5 KO, *P* > 0.05; ns, not significant.

**Figure 3:**
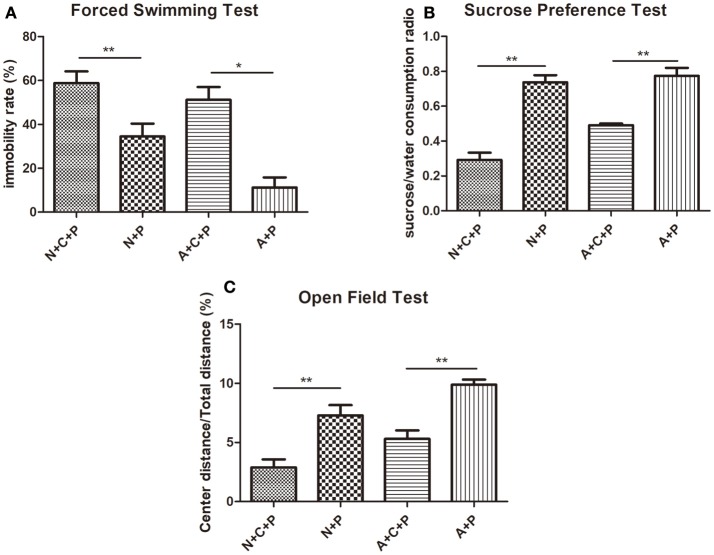
CUMS-induced depression-like behaviors. **(A)** Immobility rate of N+C+P, N+P, A+C+P, and A+P groups in FST. **(B)** Sucrose/water consumption ratio of N+C+P, N+P, A+C+P, and A+P groups in SPT. **(C)** Center distance/total distance ratio of N+C+P, N+P, A+C+P, and A+P groups in open field test (OFT). Values are expressed as mean ± SEM (*n* = 5). N+C+P vs. N+P, ^*^P < 0.05, ^**^P < 0.01; A+C+P vs. A+P, ^*^P < 0.05, ^**^P < 0.01.

Mental conditions of mice were assessed ahead of the experiment (Figures 2A,B). There was no significant difference (*P* > 0.05) in depressive-like behaviors between C57 (normal) mice and autophagy-deficient (ATG5KO) mice. After the CUMS paradigm during pregnancy, mice showed prominent depressive-like behaviors on postnatal day 7. The OFT showed the activity of mice in N+C+P and A+C+P groups to be much lower than in N+P (*P* < 0.01) and A+P (*P* < 0.01) groups (Figure 3A). Behavioral despair was measured by the immobility rate in the FST. Mice in the N+C+P group showed an increased immobility rate than that in the N+P group (*P* < 0.01), and mice in the A+C+P group showed an increased immobility rate than that in the A+P group (*P* < 0.05) (Figure 3B). In the SPT (Figure 3C), both N+C+P and A+C+P groups showed less sucrose intake than N+P and A+P groups (both *P* < 0.01).

### Fluoxetine can alleviate depressive-like behaviors

**Figure 4:**
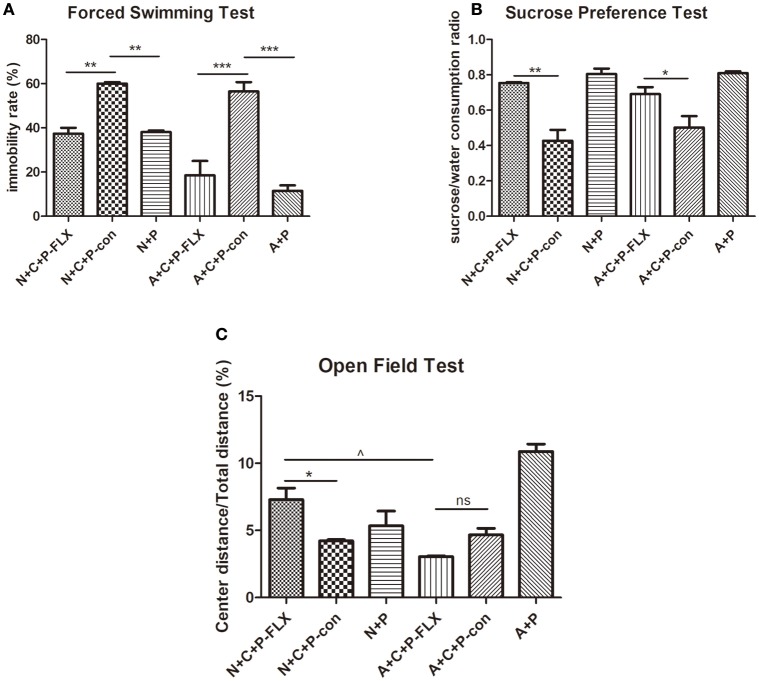
Different therapeutic effects of fluoxetine on normal and ATG5KO mice. **(A)** Immobility rate of N+C+P-FLX, N+C+P-con, N+P, A+C+P-FLX, A+C+P-con, and A+P groups in FST. **(B)** Sucrose/water consumption ratio of N+C+P-FLX, N+C+P-con, N+P, A+C+P-FLX, A+C+P-con, and A+P groups in SPT. **(C)**. Center distance/total distance ratio of N+C+P-FLX, N+C+P-con, N+P, A+C+P-FLX, A+C+P-con, and A+P groups in OFT. Values are expressed as mean ± SEM (*n* = 5). N+C+P-FLX vs. N+C+P-con, ^*^P < 0.05, ^**^P < 0.01; N+C+P-con vs. N+P, ^**^P < 0.01, *P* < 0.001; A+C+P-FLX vs. A+C+P-con, ^*^P < 0.05, ^***^P < 0.001; A+C+P-con vs. A+P, ^**^P < 0.01, *P* < 0.001; N+C+P-FLX vs. A+C+P-FLX, ^∧^*P* < 0.05; ns, not significant.

On postnatal day 28, mice were again put through the OFT, FST, and SPT (Figures 4A–C). The FST showed the immobility time of N+C+P-FLX and A+C+P-FLX groups to be significantly decreased when compared to that of N+C+P-con and A+C+P-con groups respectively (*P* < 0.01, *P* < 0.001). On the contrary, the immobility time of N+C+P-con and A+C+P-con groups were significantly increased when compared with that of N+P and A+P groups (*P* < 0.01, *P* < 0.001) (Figure 4A). The consumption of sucrose consumption ratio showed an increased sucrose intake in N+C+P-FLX and A+C+P-FLX groups when compared with that in N+C+P-con and A+C+P-con groups respectively (Figure 4B) (*P* < 0.01, *P* < 0.05). There was a significant difference in the center/total distance ratio in N+C+P-FLX and A+C+P-FLX groups (*P* < 0.05). OFT results also showed a significant difference between the N+C+P-FLX and N+C+P-con groups. (*P* < 0.05) (Figure 4C).

### Upregulation of autophagy-related proteins following fluoxetine treatment

**Figure 5:**
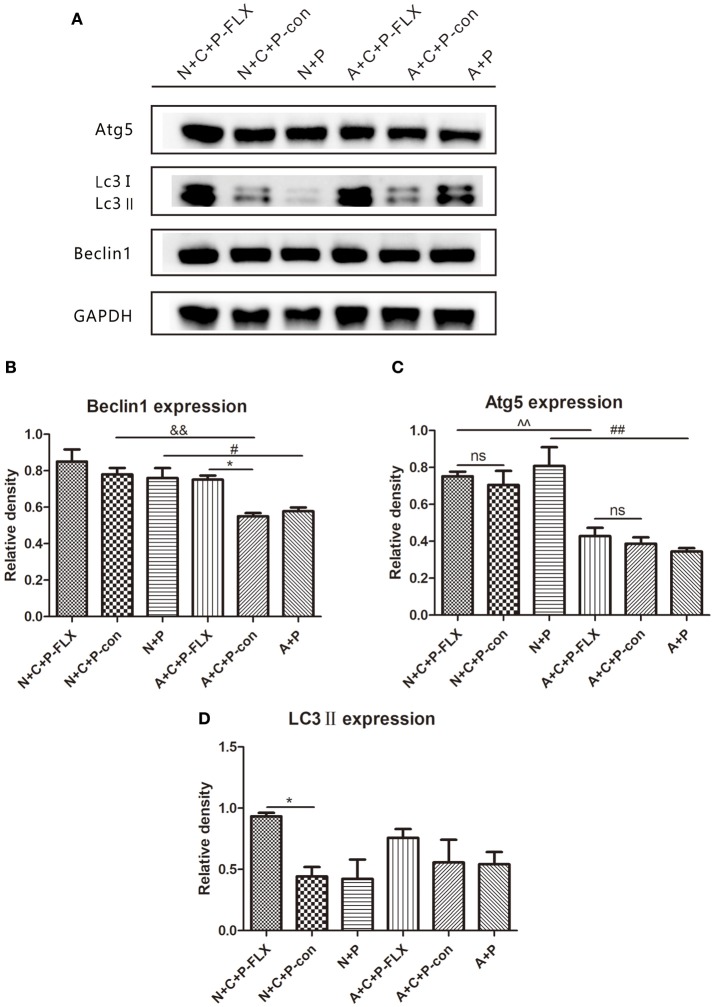
Autophagy-related proteins were upregulated after fluoxetine treatment. **(A)** Western blot results of Atg5, LC3II, Beclin1, and GADPH. Quantification analysis of **(B)** Beclin1, **(C)** Atg5, and **(D)** LC3II. Values are expressed as mean ± SEM (*n* > 5). N+C+P-FLX vs. N+C+P-con, ^*^P < 0.05; A+C+P-FLX vs. A+C+P-con, ^*^P < 0.05; N+C+P-FLX vs. A+C+P-FLX, ^∧∧^*P* < 0.01; N+C+P-con vs. A+C+P-con, ^&&^*P* < 0.01; N+P vs. A+P, ^#^*P* < 0.05, ^##^*P* < 0.01; ns, not significant.

The representative images of Western blot of Beclin-1, Atg5 and LC3II can be seen in Figure 5A. The gray value of Beclin-1 in the A+C+P-FLX group was significantly increased when compared with that in the A+C+P-con group (*P* < 0.05); however, it was lower than that in the N+C+P-FLX group. The expression of Beclin-1 in both N+C+P-con and N+P groups was significantly increased when compared with A+C+P-con and A+P groups (*P* < 0.05). (Figure 5B). Atg5 expression in both N+C+P-FLX and N+P groups was significantly higher than in A+C+P-FLX and A+P groups (*P* < 0.01); nonetheless, there were no significant changes after fluoxetine treatment (Figure 5C). LC3II showed significant increase after fluoxetine treatment in the N+C+P-FLX group in comparison to that in the N+C+P-con group (*P* < 0.05), whereas in the A+C+P-FLX group, there was an increment but it was not significant when compared to that in A+C+P-con (*P* > 0.05) (Figure 5D).

### NF-κB, iNOS, and IL-10 changes showed reduced inflammation after fluoxetine treatment

**Figure 6:**
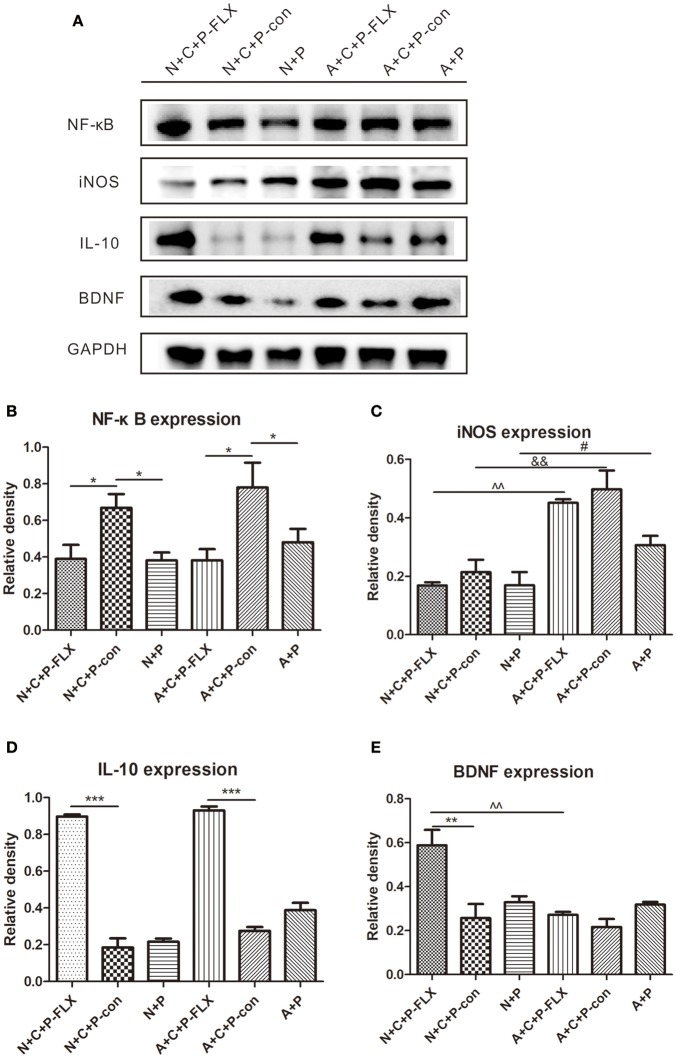
Effect of fluoxetine treatment on BDNF and inflammation levels. **(A)** Western blot results of NF-κB, IL-10, iNOS, BDNF, and GADPH. Quantification analysis of **(B)** NF-κB, **(C)** iNOS, **(D)** IL-10, and **(E)** BDNF. Values are expressed as mean ± SEM (*n* > 5). N+C+P-FLX vs. N+C+P-con, ^*^P < 0.05, ^**^P < 0.01, ^***^P < 0.001; N+C+P-con vs. N+P, ^*^P < 0.05; A+C+P-FLX vs. A+C+P-con, ^*^P < 0.05, ^***^P < 0.001; A+C+P-con vs. A+P, ^*^P < 0.05; N+C+P+FLX vs. A+C+P-FLX, ^∧∧^*P* < 0.01; N+C+P-con vs. A+C+P-con, ^&&^*P* < 0.01; N+P vs. A+P, ^#^*P* < 0.05.

NF-κB and iNOS are both key regulators of inflammatory immune responses and IL-10 can inhibit NF-κB activity. NF-κB expression in N+C+P-FLX and A+C+P-FLX groups was significantly lower than in N+C+P-con and A+C+P-con groups (*P* < 0.05). However, NF-κB expression in N+C+P-con and A+C+P-con groups was significantly increased when compared to that in N+P and A+P groups (*P* < 0.05) (Figures 6A,B). There was a significant difference in iNOS expression between A+C+P-FLX and N+C+P-FLX groups (*P* < 0.01). The iNOS expression in the N+C+P-FLX group was significantly lower than that in the A+C+P-FLX group, with both lower when compared to iNOS expression in N+C+P-con and A+C+P-con groups respectively (*P* < 0.01, *P* < 0.05) (Figures 6A,C). In contrast to iNOS, IL-10 expression was significantly increased after fluoxetine administration in both N+C+P-FLX and A+C+P-FLX groups when compared to N+C+P-con and A+C+P-FLX groups respectively (both *P* < 0.001) (Figure 6D).

### BDNF expression was upregulated following fluoxetine treatment

Following fluoxetine administration, BDNF expression in the N+C+P-FLX group showed a significant increase in comparison to that in the A+C+P-FLX group (*P* < 0.01). BDNF expression in the N+C+P-FLX group was also significantly increased when compared to that in the N+C+P-con group (*P* < 0.01) (Figures 6A,E).

### Alterations in microglia numbers following CUMS and fluoxetine treatment

**Figure 7:**
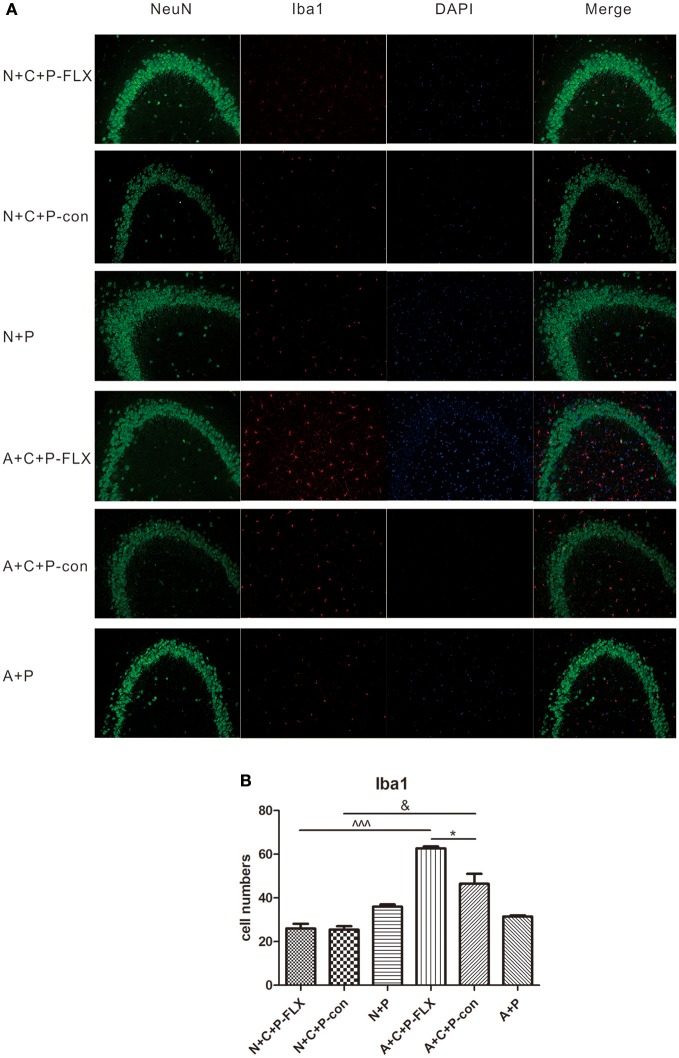
Immunofluorescence results indicated variation of microglia activation after CUMS and fluoxetine treatment. **(A)** Representative images of Iba1 (red), NeuN (green), and DAPI (blue). Merged images are shown in the right panel. Bar = 50 μm. **(B)** Quantification analysis of Iba1-positive cells. Values are expressed as mean ± SEM (*n* > 5). N+C+P-con vs. A+C+P-con, ^&^*P* < 0.01; N+C+P-FLX vs. A+C+P-FLX, ^∧∧∧^*P* < 0.001, A+C+P-FLX vs. A+C+P-con, ^*^P < 0.05.

Microglia activation can be measured by positive microglia numbers of Iba1 staining. Positive microglia (Iba1-positive) in the N+C+P-FLX group was significantly less than in A+C+P-FLX mice (^∧∧∧^*P* < 0.001). Iba1-positive microglia in the N+C+P-con group was also significantly lower than in the A+C+P-con group (^∧^*P* < 0.05), and it was significantly increased in the A+C+P-FLX group when compared with the A+C+P-con group (^∧^*P* < 0.05). Immunofluorescence detection also showed neuronal and microglial colocation alterations. Microglia showed more colocations with neurons in the dentate gyrus of the hippocampus after fluoxetine treatment in the A+C+P-FLX group when compared to the N+C+P-FLX group (Figures 7A,B).

## Discussion

In normal mice, gestating CUMS induces postpartum depressive-like behavior. This can be reversed by fluoxetine administration. Microglia-specific autophagy-deficient mice (Cx3Cr1^Cre/+^ATG5^loxp/loxp^) demonstrated lowered activity when compared to normal mice. This difference can also be found in microglia and inflammation activation. Autophagic and BDNF changes were measured for each group. Finally, we found that autophagy inhibition in microglia played an important role in regulating inflammation and BDNF, further attenuating fluoxetine treatment.

Microglia-specific autophagy-deficient mice were established with the Cre-loxP system by mating Cx3Cr1^Cre/+^ male with ATG5^loxp/loxp^ female. In the CNS, microglial cells are characterized by a high expression of the chemokine receptor CX3CR1 (21). The Atg12–Atg5 conjugate contributes to the expansion of the autophagosomal membrane, which is important to the formation of the autophagolysosome (22). Autophagic protein expressions in the hippocampus measured by the western blot had results showing significant decreases in ATG5 (Figure 5C) and subsequently substantiating *atg5* to be successfully knocked out in microglia. The CUMS model is widely used in depression studies, with the two main methods of the depression model including stress during pregnancy (7, 22, 23) and stress prior to pregnancy (4). Maternal exposure to stress has long-lasting, dissociable effects (7). Behavioral tests (FST and SPT) confirmed the mental condition of mice before the experiment (Figures 2A,B). The OFT results reflect the mice's mobility and exploration capacity. On postnatal day 7, the activity of the N+C+P and A+C+P groups after CUMS was significantly decreased when compared to control groups (Figure 3A). Meanwhile, the FST and the SPT were performed. Following CUMS, the N+C+P, and A+C+P groups showed behavioral despair and anhedonia respectively (Figures 3B,C).

Fluoxetine, a traditional antidepressant targeting serotonin reuptake inhibition, is used as treatment for depression including that of women with PPD (24–26) and requires at least 3 weeks to take effect (27). Other antidepressants like ketamine may have limited preventative benefits in PPD (28). On postnatal day 28 after fluoxetine administration for 3 weeks, behavioral tests confirmed the reversal of depressive-like behaviors. The FST showed that immobility rate was decreased, indicating a reversion in behavioral despair (Figure 4A). Microglia-specific autophagy-deficient mice showed lowered activity in comparison to normal mice. The OFT evidenced the center/total distance ratio to be increased in the N+C+P-FLX group while the ratio decreased in the A+C+P-FLX group (Figure 4B). The SPT showed the sucrose intake of the N+C+P-FLX group to be increased, indicating anhedonia alleviation (Figure 4C). The underlying mechanism of behavioral performance can be explained at the molecular level. Alcocer-Gómez posited that antidepressants containing fluoxetine show autophagy dependent-NLRP3-inflammasome inhibition in major depressive disorders (29). Fluoxetine can mitigate NLRP3 inflammasome activation through autophagy activation (30). In our study, Beclin-1 expression in A+C+P-FLX was increased (Figure 5B). LC3II expression was significantly upregulated in the N+C+P-FLX group when compared to the N+C+P-con group, demonstrating that fluoxetine can activate autophagy-related pathways (Figure 5D). Endogenous and exogenous stimuli both arouse disorders of microenvironmental homeostasis in the CNS, with microglia critically determining the fate of other neural cells (31, 32). Microglial autophagy plays an important role in the inflammation and survival of microglia. However, as to whether the activation of microglia autophagy is proinflammatory or anti-inflammatory remains ambiguous (24). Garfield and his colleagues had confirmed that PPD is most often accompanied by an elevated inflammation level (33). In this study, following fluoxetine administration, inflammatory factors such as iNOS and NF-κB were significantly upregulated while anti-inflammatory factors (IL-10) were reduced (Figures 6B–D). Inflammatory factor alterations in this study thus suggest that the activation of microglia autophagy is anti-inflammatory. Fluoxetine administration, in this study, did not induce any significant changes in both N+C+P-FLX and A+C+P-FLX groups when compared to N+C+P-con and A+C+P-con groups (Figures 6B–D). This can be explicated via the time-dependent effect of inflammatory reaction as inflammatory factors often act in a short time (34). Microglia activity was observed via Iba1 staining. Both the active and resting states of microglia were labeled. Microglia numbers in the A+C+P-FLX group was increased (Figure 7B), explicating that the repression of autophagy in microglia culminates in microglia being activated by fluoxetine administration. Overactivation of microglia can be noxious to neurons and aggravate PPD.

The association between PPD and the elevated inflammatory level has been established (35). Stressful events during pregnancy can affect the BDNF expression level in the hippocampus, thus inducing the depressive phenotype. Fluoxetine can increase BDNF expression (36). Gao found that a close relationship exists between decreased BDNF serum levels and PPD development (37). BDNF and its receptor (TrkB) in the hippocampus play a key role in PPD pathology (38). Our results evince the BDNF expression level in the N+C+P-FLX group to be increased after fluoxetine administration. However, there was no significant difference between A+C+P-FLX and A+C-P-con groups because the inhibited autophagy in microglia affects the inflammation level, which can be reflected by iNOS. BDNF expression in these two groups was both decreased compared to control, demonstrating that fluoxetine treatment was influenced by the inflammation level. On the contrary, microglia-specific autophagy-deficient mice (A+C+P-FLX group) did not present an increment in BDNF expression (Figures 6A,E). This corroborates that autophagy deficiency in microglia has an adverse effect on PPD treatment via elevated inflammation.

Although our study results address how fluoxetine influences BDNF expression, downstream signals require further research. We intend to investigate the time effect of fluoxetine treatment in different postpartum periods in our future studies. We will also attempt to probe into autophagic flux alterations during depression development and its relationship with neurotransmitters. Our research provides microglia autophagy as a new target for the clinical treatment of PPD.

## Author contributions

MF designed the experiments. XT drafted the manuscript. XT, XD, and YJ performed the experiments, analyzed the data. ZH and BB revised the manuscript. All authors read and approved the final manuscript.

### Conflict of interest statement

The authors declare that the research was conducted in the absence of any commercial or financial relationships that could be construed as a potential conflict of interest. The handling Editor declared a shared affiliation, though no other collaboration, with one of the authors with several of the authors XT, XD, YJ, BB, MF at the time of the review.
